# Two−Dimensional Planar Penta−NiPN with Ultrahigh Carrier Mobility and Its Potential Application in NO and NO_2_ Gas Sensing

**DOI:** 10.3390/mi14071407

**Published:** 2023-07-12

**Authors:** Hao Wang, Gang Li, Jun-Hui Yuan, Jiafu Wang, Pan Zhang, Yahui Shan

**Affiliations:** 1 Wuhan Second Ship Design and Research Institute, Wuhan 430205, China; 2 College of Railway Rolling Stock, Wuhan Railway Vocational College of Technology, Wuhan 430205, China; 3 School of Science, Wuhan University of Technology, Wuhan 430070, China; 4 School of Integrated Circuits, Peking University, Beijing 100871, China; panzhangpku@gmail.com

**Keywords:** two−dimensional materials, penta−NiPN monolayer, carrier mobility, gas sensor, first−principles calculations

## Abstract

Two−dimensional (2D) materials with novel structures and electronic properties are promising candidates for the next generation of micro− and nano−electronic devices. Herein, inspired by the recent experimental synthesis of penta−NiN_2_ (*ACS Nano*, 2021, 15, 13539–13546), we propose for the first time a novel ternary penta−NiPN monolayer with high stability by partial element substitution. Our predicted penta−NiPN monolayer is a quasi−direct bandgap (1.237 eV) semiconductor with ultrahigh carrier mobilities (10^3^–10^5^ cm^2^V^−1^s^−1^). Furthermore, we systematically studied the adsorption properties of common gas molecules (CO, CO_2_, CH_4_, H_2_, H_2_O, H_2_S, N_2_, NO, NO_2_, NH_3_, and SO_2_) on the penta−NiPN monolayer and its effects on electronic properties. According to the energetic, geometric, and electronic analyses, the penta−NiPN monolayer is predicted to be a promising candidate for NO and NO_2_ molecules. The excellent electronic properties of and the unique selectivity of the penta−NiPN monolayer for NO and NO_2_ adsorption suggest that it has high potential in advanced electronics and gas sensing applications.

## 1. Introduction

Sensors are electronic devices designed to detect and perceive their surroundings or specific substances, such as gas, light, temperature, humidity, the human body, etc., and they received significant concern because of their importance to the information society [1,2,3]. They can convert analog signals into digital signals, then send them to the central processor for processing, and finally realize the detection of the calibration object. Due to their importance and irreplaceability in today’s society, the development of new sensors is a popular research topic.

Currently, gas sensors are among the most widely studied type of sensors. High−performance gas sensors are in great demand for industrial production, environmental monitoring, and clinical medicine. Two−dimensional (2D) materials have emerged as promising gas−sensitive materials due to their large surface area, high surface activity, and abundant surface active sites [4,5,6,7]. For example, Ou et al. proved that 2D SnS_2_ is a potential material for selective and reversible NO_2_ detection at low operating temperatures [8], while Qin et al. developed a flexible paper substrate sensor based on 2D WS_2−x_ for NH_3_ detection at room temperature [9]. So far, many types of gas detection have been realized experimentally or theoretically based on various 2D materials, such as NO, CO, SO_2_, NH_3,_ H_2_O, and others [10,11,12,13]. However, developing a high−sensitivity, fast−response, and completely desorbed gas sensor based on 2D materials under harsh working conditions remains a challenge. Nonetheless, the search for new 2D materials with excellent electronic properties that can be used in gas sensors remains a major research focus.

Recently, Bykov et al. [14] successfully experimentally realized penta−NiN_2_ with an ideal Cairo tessellation via a high−pressure route. Notably, this penta−NiN_2_ has a typical layered structure and is stable at room temperature, suggesting that a penta−NiN_2_ monolayer can be obtained by mechanical exfoliation, similar to that with graphene or black phosphorene [15,16]. Theoretical studies have shown that the penta−NiN_2_ monolayer is a direct bandgap semiconductor with moderate thermal conductivity and outstanding mechanical properties, and it can be applied to bifunctional oxygen electrocatalysts and gas sensors [17,18,19,20,21]. In fact, before the experimental synthesis of penta−NiN_2_, similar structures had been studied extensively, such as penta−MX_2_ (M = Ni, Pd, Pt; X = N, P, As, Sb) [17,22,23,24,25,26,27]. This family of 2D materials demonstrated excellent properties, such as a suitable bandgap, a high optical absorption coefficient, ultrahigh carrier mobility, and so on [22,23,24,25]. However, most research on this family of 2D materials focused on binary systems, and previous studies showed that the properties of systems can be effectively improved by introducing homologous elements, such as α−P_1−x_As_x_ [28]. Therefore, the successful experimental realization of penta−NiN_2_ provides a material basis for further study of relevant ternary systems.

In this work, we predicted a new planar ternary penta−NiPN monolayer by atomic substitution of the penta−NiN_2_ monolayer using first−principles calculations. After careful stability analysis, we focused on electronic structures and adsorption properties for 12 kinds of common gases. Electronic property analysis showed that the penta−NiPN monolayer is a quasi−direct bandgap semiconductor (1.237 eV) with ultrahigh carrier mobility (up to 10^5^ cm^2^V^−1^s^−1^). The results of gas adsorption revealed that the penta−NiPN monolayer exhibits good selectivity for NO and NO_2_, indicating its potential as a gas−sensitive material for NO and NO_2_.

## 2. Methods

All structural optimization and electronic properties in this work were obtained via the Vienna *Ab initio* Simulation Package (VASP) [29,30], based on density functional theory (DFT) [31,32]. The cutoff energy was set to 500 eV, and the generalized gradient approximation (GGA) [33] with Perdew–Burke–Ernzerhof (PBE) functional form was used to describe the exchange–correlation energy. However, to overcome the bandgap underestimation problem of GGA–PBE for semiconductors or insulators, the hybrid functional HSE06 [34] was also employed for electronic structure calculations. Moreover, the valence electrons of different elements in the calculation were set as follows: 1*s* for H, 2*s* and 2*p* for C/N/O, 3*s* and 3*p* for P/S, and 4*d* and 5*s* for Ni. The convergence accuracy of total energy and force in the calculation was set a 10^−7^ eV and 0.005 eV/Å per atom, respectively. For geometry optimization, a 12 × 12 × 1 and a 5 × 5 × 1 *k*−point grid, following the scheme of Monkhorst–Pack [35], was used to sample the Brillouin zones for unit cells of the penta−NiPN monolayer and the gas adsorption supercell model, respectively. Denser *k*−point grids were used for self−consistent and electronic property calculations. Grimme’s DFT–D2 [36] was adopted to describe the van der Waals (vdW) corrections in gas adsorption. A vacuum layer of 20 Å length was introduced into all models to avoid interactions between adjacent layers. Phonon dispersion for the new predicted penta−NiPN monolayer was performed using VASP and Phonopy code [37], based on density functional perturbation theory (DFPT). Additionally, ab initio molecular dynamics (AIMD) simulations lasting for 5 ps at room temperature were employed to evaluate the thermal stability of the penta−NiPN monolayer using a 4 × 4 × 1 supercell.

## 3. Results and Discussion

### 3.1. Structure and Stability

**Figure 1:**
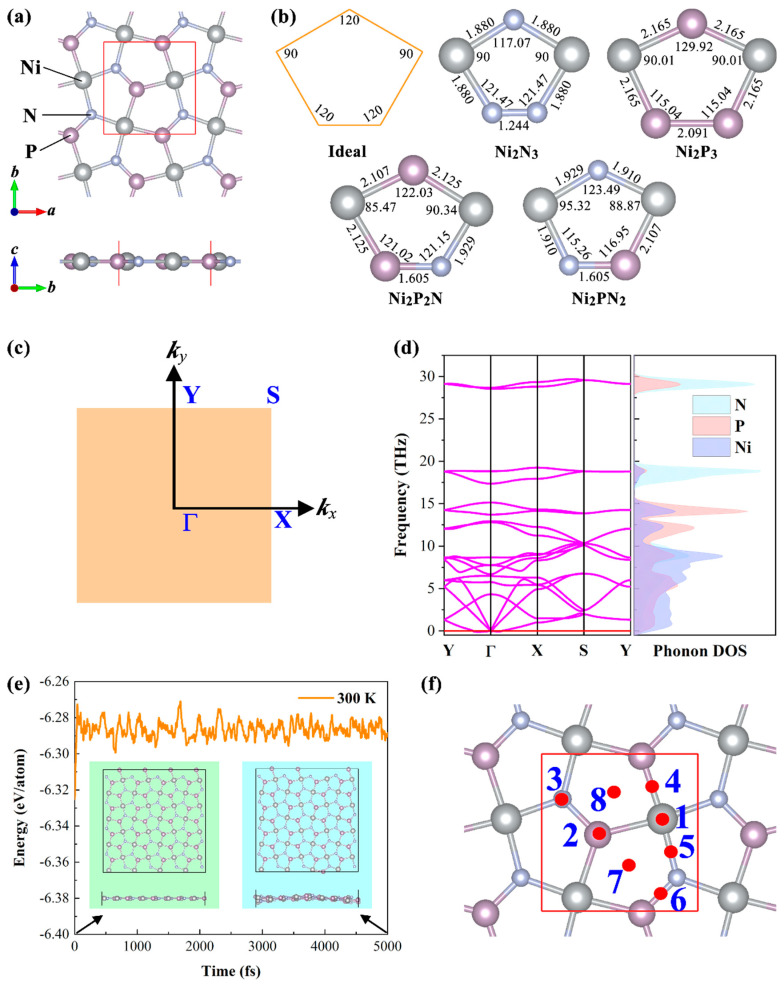
(**a**) Top and side views of the penta−NiPN monolayer; the unit cell is marked by a red border. (**b**) Geometry of single ideal (pentagonal Cairo tiling) Ni_2_N_3_, Ni_2_P_2_N, Ni_2_PN_2_, and Ni_2_P_3_ pentagons. Numbers indicate interatomic distances (Å) and bond angles (deg). (**c**) The Brillouin zone of the penta−NiPN monolayer. (**d**) The phonon dispersion and corresponding phonon DOS of the penta−NiPN monolayer. (**e**) The AIMD simulation results of the penta−NiPN monolayer at 300 K; the insert is the initial and final structure of the penta−NiPN monolayer. (**f**) The eight adsorption sites have been labeled in the diagram.

The penta−NiPN monolayer, shown in Figure 1a, is a perfect single atomic thickness planar 2D material, similar to graphene. The penta−NiPN monolayer consists of Ni_2_P_2_N pentagons and Ni_2_PN_2_ pentagons with a calculated lattice constant *a*/*b* of 4.995/5.011 Å (see Table 1). This value is larger than that of the penta−NiN_2_ monolayer (4.53 Å) [17] and smaller than that of the penta−NiP_2_ monolayer (5.55 Å) [22]. The penta−NiPN monolayer has lower symmetry (space group: *Pb*2_1_*m*, No.26) than a single element system (space group: *P*4/*mbm*, No.127) due to the existence of two non−metallic elements (N and P). As a result, two kinds of unequal Ni−N bonds and Ni−P bonds with bond lengths of 1.929/1.910 Å and 2.125/2.107 Å, respectively, exist in the penta−NiPN monolayer. The bond lengths in the penta−NiPN monolayer are between the lengths of the Ni−N and Ni−P bonds in the penta−NiN_2_ monolayer (1.88 Å) and the penta−NiP_2_ monolayer (2.16 Å). The N−P bond length in the penta−NiPN monolayer is 1.605 Å, while the N−N bond length and P−P bond length in penta−NiN_2_ monolayer and penta−NiP_2_ monolayer are 1.24 Å and 2.11 Å, respectively.

The ideal pentagonal Cairo tiling maintains bond angles of 90° and 120°, as depicted in Figure 1b. It is clear that pentagonal monolayers consisting of two (e.g., penta−NiN_2_ and −NiP_2_) or three elements cannot form a perfect pentagonal Cairo tile. Figure 1b shows the corresponding bond lengths and bond angles of Ni_2_N_3_, Ni_2_P_2_N/Ni_2_PN_2_, and Ni_2_P_3_ pentagons in the penta−NiN_2_, NiPN, and NiP_2_ monolayers. For the Ni_2_N_3_ and Ni_2_P_3_ pentagons, the corresponding bond angles of N−Ni−N (90°) and P−Ni−P (90.01°) coincide with the ideal pentagonal Cairo tile. However, among other bond angles, such as Ni−N(P)−N(P) and Ni−N(P)−Ni, the results largely deviate from the ideal pentagonal Cairo tile. Among pentagonal monolayers, the penta−NiPN monolayer is notably more complex than the penta−NiN_2_ and −NiP_2_ monolayers. Due to the introduction of two non−equivalent non−metallic elements, there exist non−equivalent pentagons (Ni_2_P_2_N and Ni_2_PN_2_) in the lattice (as seen in Figure 1b). Generally, the Ni_2_P_2_N and Ni_2_PN_2_ pentagons undergo varying degrees of distortion compared to the ideal pentagonal Cairo tiling. Naturally, this atomic divergence leads to an array of unique characteristics in this predicted monolayer.

Structural stability forms the foundation for all subsequent studies. We then focus on the stability of the predicted penta−NiPN monolayer, covering its kinetic, thermodynamic, and mechanical stability. Based on DFPT, we initially calculated the phonon dispersion of the penta−NiPN monolayer, as displayed in Figure 1d. The negligible imaginary frequency of the phonon spectrum (occurring near the Γ−point) is primarily due to calculation errors. Despite this fact, the kinetic stability of the penta−NiPN monolayer is unmistakable. Additionally, the phonon density of states (DOS) analysis shows that the low−frequency portion predominantly arises from Ni−P bonds, whereas the high−frequency portion is dominated by Ni−N and N−P bonds. This result indicates the robust bonding traits of the penta−NiPN monolayer. The thermodynamic stability analysis of the penta−NiPN monolayer at room temperature (300 K) was performed via ab initio molecular dynamics simulations. As shown in Figure 1e, the inherent energy fluctuation of the system, covering a simulation time of 5 ps, was within 0.025 eV/atom. No observable structural collapse occurred in the atomic structure of the final state, indicating the high thermodynamic stability of the predicted penta−NiPN monolayer at room temperature. Cohesive energy (*E*_coh_), as another thermodynamic indicator, can evaluate the predicted system’s relative realizability under experimental conditions. The *E*_coh_ of the penta−NiPN monolayer is defined as $E_{\mathrm{coh}}=\left(2E_{\mathrm{Ni}}+2E_{P}+2E_{N}-E_{\mathrm{NiPN}}\right)/6$, where $E_{\mathrm{Ni}}$/$E_{P}$/$E_{N}$ and $E_{\mathrm{NiPN}}$ are the energy of a single Ni/P/N atom and the total energy of penta−NiPN monolayer, respectively. According to the definition, a higher *E*_coh_ value signifies greater stability. The calculated *E*_coh_ of the penta−NiPN monolayer is 4.55 eV, which is higher than that of the NiP_2_ monolayer (4.09 eV, 3.944 eV) [22,23], silicene (3.94 eV) [24], and phosphorene (3.477 eV) [38] but slightly lower than that of penta−NiN_2_ (4.98 eV) [17]. As silicene and phosphorene have been obtained successfully in experiments, the high−realization potential of the penta−NiPN monolayer is likewise promising, especially considering that Bykov et al. [14] recently achieved a room−temperature stable penta−NiN_2_ layer experimentally. Therefore, it is reasonable to anticipate the experimental realization of a penta−NiPN monolayer (such as via element substitution doping) in the near future.

In addition to kinetics and thermodynamic stability, mechanical stability is another critical factor to consider for newly predicted monolayers. According to the Born–Huang criterion [39], stable 2D materials should satisfy:$C_{11}C_{22}-C_{12}^{2}>0$ and $C_{66}>0$, where *C*_11_, *C*_22_, *C*_12_, and *C*_66_ are the independent elastic constants of the predicted monolayer. For the penta−NiPN monolayer, the calculated *C*_11_, *C*_22_, *C*_12_, and *C*_66_ are 158.25 N m^−1^, 154.64 N m^−1^, 31.85 N m^−1^, and 41.22 N m^−1^, respectively. These values confirm that the predicted penta−NiPN monolayer in this work possesses good mechanical stability.

**Figure 2:**
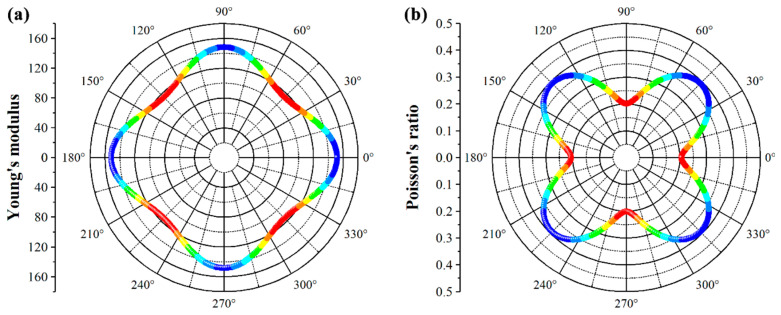
Angle−dependent (**a**) Young’s modulus and (**b**) Poisson’s ratio of the penta−NiPN monolayer.

Furthermore, Young’s modulus (*Y*) and Poisson’s ratio (*υ*) are important indices for measuring the mechanical properties of materials. We further evaluate these properties for the predicted penta−NiPN monolayer using the calculated independent elastic constants (*C*_11_, *C*_22_, *C*_12_, and *C*_66_) above. The angle dependent in−plane Young’s modulus *Y*(*θ*) and Poisson’s ratio *υ*(*θ*) can be expressed as follows [40]:

(1) $$Y(\theta )=\frac{C_{11}C_{12}-C_{12}^{2}}{C_{11}\sin^{4}\theta +A\sin^{2}\theta \cos^{2}\theta +C_{22}\cos^{4}\theta }$$

(2) $$\upsilon (\theta )=\frac{C_{12}\sin^{4}\theta -B\sin^{2}\theta \cos^{2}\theta +C_{12}\cos^{4}\theta }{C_{11}\sin^{4}\theta +A\sin^{2}\theta \cos^{2}\theta +C_{22}\cos^{4}\theta }$$

where $A=(C_{11}C_{22}-C_{12}^{2})/C_{66}-2C_{12}$ and $B=C_{11}+C_{22}-(C_{11}C_{22}-C_{12}^{2})/C_{66}$. As shown in Figure 2, the Young’s modulus and Poisson’s ratio of the penta−NiPN monolayer are anisotropic and exhibit angle dependence, different from those of the penta−NiN_2_ and −NiP_2_ monolayer. The maximum Young’s modulus is 151.69 N m^−1^ along the *x* direction (*Y*_11_, *θ* = 0°/180°), while the *Y*_22_ (148.23 N m^−1^, *θ* = 90°/270°) is slightly smaller than that of *Y*_11_. The minimum Young’s modulus is 114.66 N m^−1^ along the diagonal direction (*θ* = 45°/135°/225°/315°). The Young’s modulus of the penta−NiPN monolayer is lower than that of the penta−NiN_2_ monolayer (168.8 N m^−1^) [17], but higher or comparable to that of the penta−NiP_2_ monolayer (122.19 N m^−1^) [22]. The corresponding Poisson’s ratio of the penta−NiPN monolayer is shown in Figure 2b. In contrast to the Young’s modulus, the minimum value of Poisson’s ratio is obtained in the axial direction *υ*_22_ (*θ* = 90°/270°), where the value is 0.201, while *υ*_11_ (0.206, *θ* = 0°/180°) is slightly higher than *υ*_22_. In addition, the penta−NiPN monolayer has a maximum Poisson’s ratio value of 0.391 in the diagonal direction (*θ* = 45°/135°/225°/315°). The minimum Poisson’s ratio of the penta−NiPN monolayer is much higher than that of the penta−NiN_2_ monolayer (0.130) [17] but comparable to that of the penta−NiP_2_ monolayer (0.22). Therefore, in general, the penta−NiPN monolayer is less stiff and more flexible than the penta−NiN_2_ and −NiP_2_ monolayers. With the intrinsic anisotropy of the penta−NiPN monolayer considered, it can be anticipated that penta−NiPN has a more diverse and adjustable set of mechanical properties than the penta−NiN_2_ and −NiP_2_ monolayers.

In short, the penta−NiPN monolayer is predicted to be robust and stable, providing a strong theoretical basis for its design, and it has also been shown to be feasible for experimental investigation. Its diverse structural and mechanical properties make it a highly promising candidate for use as a 2D functional material.

### 3.2. Electronic Structure

**Figure 3:**
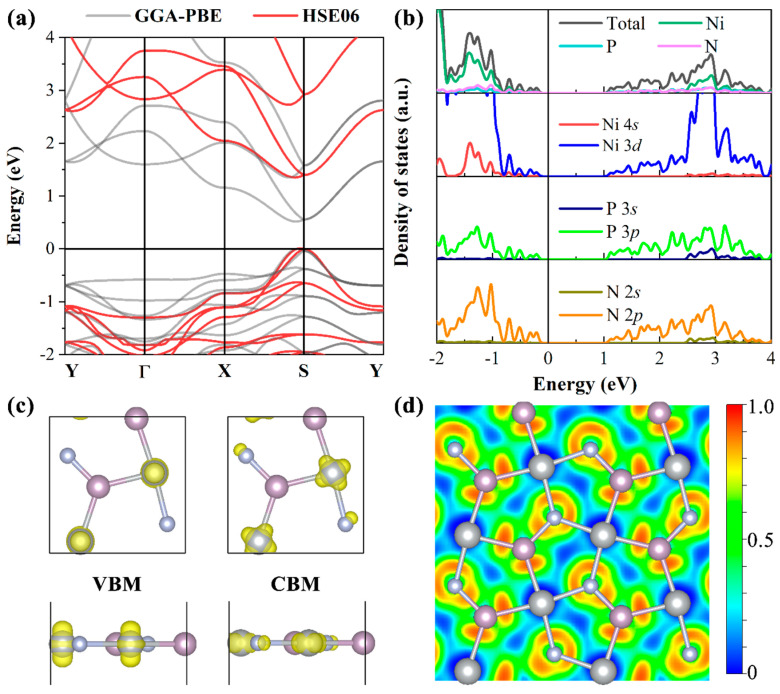
Calculated (**a**) electronic band structures and (**b**) DOS, as well as PDOS, of the penta−NiPN monolayer at the HSE06 level. (**c**) Spatial distributions of the wave functions corresponding to the VBM and CBM of the penta−NiPN monolayer at the GGA–PBE level. The charge contour density is 0.01 *e*/Å^3^. (**d**) Calculated ELF of the penta−NiPN monolayer.

Figure 3a,b shows the electronic band structures and density of states (DOS) with partial density of states (PDOS) of the penta−NiPN monolayer. The electronic band structures are presented using both the standard GGA–PBE (solid gray line) and hybrid functional HSE06 (solid red line). For the DOS and PDOS, only the results at the HSE06 level are presented. The calculated band morphologies based on GGA–PBE and HSE06 are almost identical. Unlike the penta−NiN_2_ and −NiP_2_ monolayers, which exhibit direct bandgap features, the penta−NiPN monolayer is an indirect bandgap semiconductor with a bandgap value of 0.518/1.237 eV at the PBE/HSE06 level. As summarized in Table 1, the bandgap of the penta−NiPN monolayer is larger than that of the penta−NiN_2_ (1.10 eV) and −NiP_2_ (0.81 eV) monolayers. The valence band maximum (VBM) and conduction band minimum (CBM) of the penta−NiPN monolayer are both located near the S−point along the S−X direction, whereas for the penta−NiN_2_ and −NiP_2_ monolayers, the VBM and CBM are both located at the S−point. It should be noted that, although the penta−NiPN monolayer is an indirect bandgap semiconductor, the energy difference (Δ*E*) between its direct and indirect bandgaps is only 0.0013/0.0449 eV at the PBE/HSE06 level, making it a quasi−direct bandgap semiconductor. The combination of a suitable bandgap and quasi−direct band features in the penta−NiPN monolayer may have potential applications in optoelectronic devices.

As shown in Figure 3b, the PDOS results indicate that there is strong hybridization between the Ni 3*d* orbits and P 3*p* and N 2*p* orbits. Furthermore, the orbital contributions to VBM and CBM are dominated by Ni 3*d* orbits, which is further confirmed by the spatial distribution of the wave−functions corresponding to the VBM and CBM of the penta−NiPN monolayer at the GGA–PBE level (see Figure 3c). The spatial distribution of the wave functions of the VBM is mainly located around Ni atoms, while that of CBM is located around Ni atoms and N atoms. Regarding bonding properties, as shown in Figure 3d, the electron localization function (ELF) [41,42] reveals that Ni−N and Ni−P are typical ionic bonds with the electron mainly located around the non−metal element. The N−P bond is mainly an ionic bond with some covalent bond characteristics. The results of Bader charge analysis [43] indicate that both Ni and P lose electrons during the bonding process of the penta−NiPN monolayer, with −0.6848 *e*/atom and −1.5487 *e*/atom, respectively, while N gains +2.2335 *e*/atom (“+” and “−” representing gained and lost electrons, respectively). The charge transfer during the bonding process is mainly determined by the electronegativity of each element. The corresponding electronegativities of Ni, P, and N are 1.91, 2.19, and 3.04, respectively. As a result, N gains the most electrons during the bonding process, as expected.

**Figure 4:**
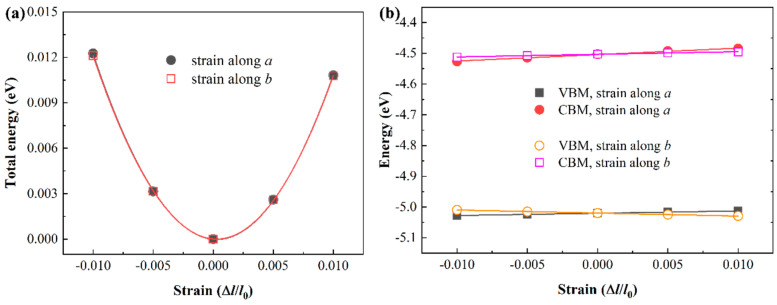
(**a**) The relationship between the total energy and the applied strain (Δ*l*/*l*_0_) along the *a*/*b* direction of the penta−NiPN monolayer. (**b**) The shift of VBMs and CBMs for the penta−NiPN monolayer with respect to the vacuum energy under the applied strain along the *a*/*b* direction. All the calculations are at the GGA–PBE level.

To evaluate the potential of the penta−NiPN monolayer for electronic devices, we calculated its acoustic phonon−limited carrier mobility, a critical parameter in this regard. Since the band morphologies of the monolayer are almost identical at the GGA–PBE and HSE06 levels, we used the GGA–PBE results directly to evaluate the carrier mobility here. The method used is based on the deformation potential theory (DPT) proposed by Bardeen and Shockley [44], with further details described in numerous works [45,46], which we do not repeat here. To calculate the carrier mobility, we evaluated the carrier effective mass m^*^, the deformation potential constant |*E*_il_|, and the elastic modulus *C*_2D_ of the penta−NiPN monolayer, as summarized in Table 2. In addition, Figure 4 shows more details for the calculation of *C*_2D_ and *E*_il_. Our calculations show that the effective masses m^*^ of electrons and holes in the penta−NiPN monolayer along the *a*/*b*−direction are 0.38/0.36 *m*_e_ and 0.22/0.24 *m*_e_, respectively, which are higher than those in the penta−NiP_2_ monolayer (0.106/0.140 *m*_e_ and 0.119/0.170 *m*_e_ along the *a*/*b*−direction, respectively) [22]. Meanwhile, the deformation potential constant of the penta−NiPN monolayer is much smaller than that of penta−NiP_2_ (2.10/0.85 eV vs. 5.23/5.23 eV; 0.74/0.99 eV vs. 1.53/1.53 eV), whereas the elastic constant of the penta−NiPN monolayer is larger than that of the penta−NiP_2_ monolayer (147.68/146.24 N m^−1^ vs. 118.19/118.19 N m^−1^). By combining the effective mass, deformation potential constant, and elastic constant, we calculated the carrier mobility of penta−NiPN monolayer. The obtained electron mobilities are 0.51 and 3.24 × 10^4^ cm^2^V^−1^ s^−1^ along the *a*− and *b*−directions, respectively, whereas the hole mobility is even higher, reaching up to 11.36 and 5.76 × 10^4^ cm^2^V^−1^s^−1^ along *a*− and *b*−directions, respectively. These values are much higher than those of some typical 2D materials, such as MoS_2_ (~49 to 200 cm^2^V^−1^s^−1^) [47], GaPS_4_ (~14 to 1306 cm^2^V^−1^s^−1^) [40], and GeP_3_ (~14 to 190 cm^2^V^−1^s^−1^) [48], and comparable to that of phosphorene (~10^4^ cm^2^V^−1^s^−1^) [49] and penta−MX_2_ (M = Ni, Pd, Pt; X = N, P, As, Sb; 10^3^~10^5^ cm^2^V^−1^s^−1^) [22,23,24]. The suitable bandgap and ultra−high carrier mobility in the penta−NiPN monolayer make it promising for nanoelectronics and microelectronics.

### 3.3. Gas Adsorption

Today, one of the most significant uses of 2D materials is in gas sensors. Previous studies have shown that 2D materials with pentagonal structures are good gas−sensitive materials, such as penta−graphene [50], penta−BCN [51], and penta−BeP_2_ [52]. On the other hand, penta−NiN_2_ [21] and penta−PdAs_2_ [27], which belong to the same family as the penta−NiPN proposed in this work, have proved to be very good gas−sensitive materials as well. Therefore, we believe that the gas−sensitive properties of the penta−NiPN monolayer are worth exploring. In this section, we focus on evaluating the potential applications of the penta−NiPN monolayer in gas sensors by exploring its gas adsorption properties. First, there are eight unequal adsorption sites in the penta−NiPN monolayer, as labeled in Figure 1f. These eight adsorption sites comprise the top site of the Ni/P/N atom (site 1/2/3), a bridge site located along the Ni−P/Ni−N/N−P bond (site 4/5/6), and a hollow site present in the Ni_2_P_2_N/Ni_2_PN_2_ pentagon (site 7/8). For gases, we selected 12 typical gas molecules as the study objects, i.e., CO, CO_2_, CH_4_, H_2_, H_2_O, H_2_S, N_2_, NO, NO_2_, NH_3_, SO_2_, and O_2_. Our main reason for choosing H_2_O and O_2_ was to evaluate the moisture sensitivity and oxidation resistance of the penta−NiPN monolayer, respectively. The remaining gases included greenhouse gases, toxic gases, or gases commonly found in the air.

**Figure 5:**
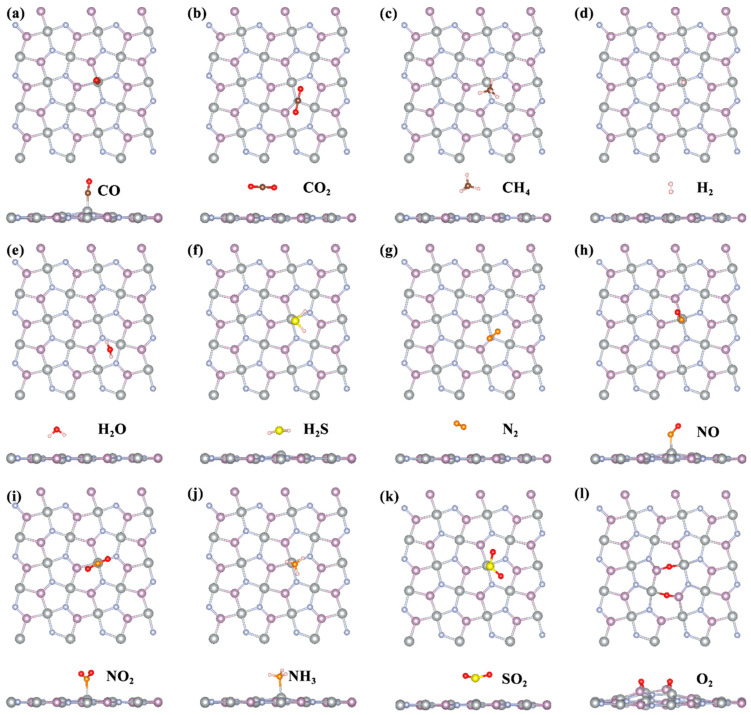
Top and side views of the most favorable adsorption configurations for (**a**) CO, (**b**) CO_2_, (**c**) CH_4_, (**d**) H_2_, (**e**) H_2_O, (**f**) H_2_S, (**g**) N_2_, (**h**) NO, (**i**) NO_2_, (**j**) NH_3_, (**k**) SO_2_, and (**l**) O_2_ on the penta−NiPN monolayer.

To identify the optimal adsorption sites of the 12 gas molecules on the penta−NiPN monolayer, we constructed 3 × 3 supercells (area: 14.98 Å × 15.03 Å). Figure 5 illustrates the most favorable adsorption configurations for the selected 12 molecules on the penta−NiPN monolayer via top and side views. For most gas molecules, the preferred adsorption site was the top site of the Ni atom, including CO, H_2_, H_2_S, NO, NO_2_, NH_3_, and SO_2_. For CO_2_ and N_2_, the top site of the N atom was more favorable. CH_4_ tended to be adsorbed at the bridge site along the Ni−N bond, while H_2_O was adsorbed at the hollow site of the Ni_2_PN_2_ pentagon. The most unusual case was during the adsorption of O_2_ on the penta−NiPN monolayer, whereby it reacted directly with the substrate, leading to the dissociation of O_2_ molecules into O atoms and ultimately forming a new material. However, for a reusable gas−sensitive material, it is necessary to have the ability to both adsorb and release gases. According to the results of O_2_ adsorption on the penta−NiPN monolayer, this process is irreversible. Therefore, our findings indicate that the penta−NiPN monolayer may require an oxygen−free environment if used as a medium material for a gas sensor. We do not discuss the case of O_2_ in the subsequent studies, considering the strong reactivity during the adsorption of oxygen and the penta−NiPN monolayer.

**Figure 6:**
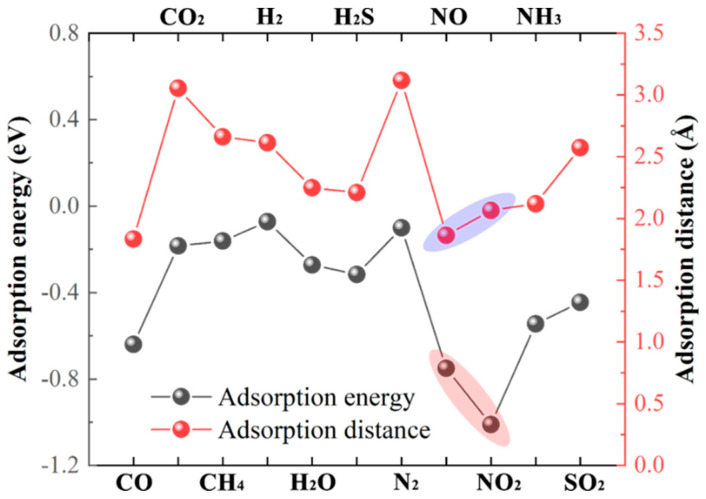
The calculated adsorption energy and adsorption distance for CO, CO_2_, CH_4_, H_2_, H_2_O, H_2_S, N_2_, NO, NO_2_, NH_3_, and SO_2_ on the penta−NiPN monolayer.

We begin our analysis of gas adsorption on the penta−NiPN monolayer with the evaluation of the adsorption energy (*E*_a_) and adsorption distance (*d*). The *E*_a_ of gas adsorption on the penta−NiPN monolayer is defined as follows: $E_{a}=E_{\mathrm{NiPN}-\mathrm{gas}}-E_{\mathrm{NiPN}}-E_{\mathrm{gas}}$, where $E_{\mathrm{NiPN}-\mathrm{gas}}$, $E_{\mathrm{NiPN}}$, and $E_{\mathrm{gas}}$ are the total energy of the NiPN monolayer with gas adsorption, a pristine NiPN monolayer, and a single gas molecule, respectively. By definition, a negative *E*_a_ implies that gas adsorption is an exothermic process and can be spontaneous. Conversely, if the *E*_a_ value is positive, the process is endothermic and non−spontaneous. The magnitude of the absolute value determines the likelihood of the reaction. The adsorption distance *d* refers to the minimum distance between the gas molecule and the substrate at the optimal adsorption site. We conducted a statistical analysis of *E*_a_ and *d* for 11 gases adsorbed on the penta−NiPN monolayer, as shown in Figure 6 and Table 3. For the 11 gases studied, the adsorption energy on the NiPN monolayer is negative (−1.011 to −0.072 eV), indicating that all adsorption could be spontaneous. Furthermore, the absolute values of adsorption energy are in the order of $\left|E_{a}^{{\mathrm{NO}}_{2}}|>|E_{a}^{\mathrm{NO}}|>|E_{a}^{\mathrm{CO}}|>|E_{a}^{{\mathrm{NH}}_{3}}|>|E_{a}^{{\mathrm{SO}}_{2}}|>|E_{a}^{H_{2}S}|>|E_{a}^{H_{2}O}|>|E_{a}^{{\mathrm{CO}}_{2}}|>|E_{a}^{{\mathrm{CH}}_{4}}|>|E_{a}^{N_{2}}|>|E_{a}^{H_{2}}\right|$. The largest was $\left|E_{a}^{{\mathrm{NO}}_{2}}\right|$ (1.011 eV), followed by $|E_{a}^{\mathrm{NO}}|\;$= 0.751 eV, indicating that the penta−NiPN monolayer is an excellent trapping material for these two gases. On the other hand, H_2_ and N_2_ exhibited very small adsorption energy values (0.072 eV and 0.100 eV), suggesting that they are challenging to capture in normal environments. Regarding the adsorption distances, the values ranged from 1.834 Å (CO) to 3.117 Å (N_2_). The adsorption distances for NO and NO_2_ are 1.862 Å and 2.065 Å, respectively, which are shorter than the values observed for NO and NO_2_ absorption on the penta−NiN_2_ monolayer (2.190 Å and 2.124 Å) [21]. Adsorption energy and distance can characterize the strength or weakness of interactions between gas molecules and host materials. Our results show that six gas molecules, including CO, H_2_S, NO, NO_2_, NH_3_, and SO_2_, had relatively strong interactions with the penta−NiPN monolayer.

**Figure 7:**
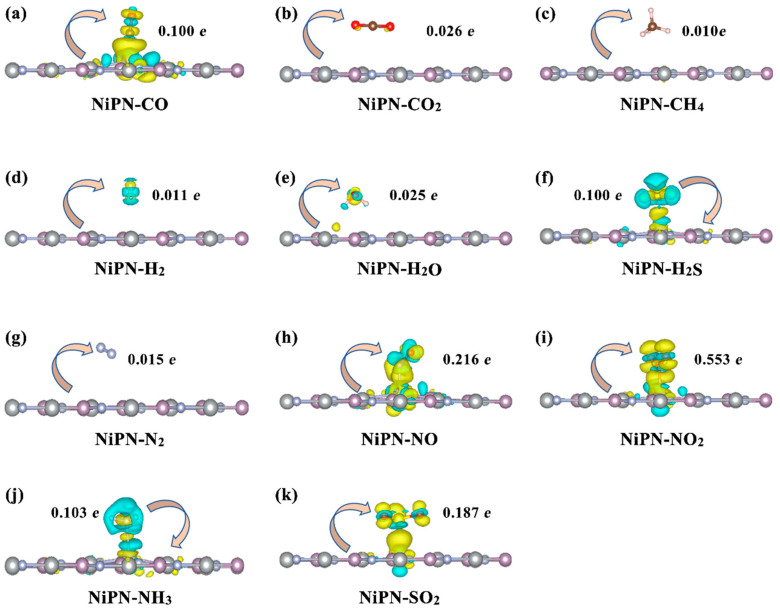
The charge density differences between (**a**) CO, (**b**) CO_2_, (**c**) CH_4_, (**d**) H_2_, (**e**) H_2_O, (**f**) H_2_S, (**g**) N_2_, (**h**) NO, (**i**) NO_2_, (**j**) NH_3_, and (**k**) SO_2_ gas molecules and the penta−NiPN monolayer. The equivalent surface was 0.012 *e*/Å^3^, and the electron accumulation (loss) is represented by yellow (blue). In addition, the direction of charge transfer (represented by arrows) and the amount of charge transfer are marked.

The study of the interaction between adsorbed gas molecules and the host material was further characterized using the charge density difference (CDD). Additionally, by conducting Bader charge analysis, we obtained the value of charge transfer (*Q*) between gas molecules and substrates. The transferred charge values for all models are summarized in Table 3, and the CDDs are plotted in Figure 7. In general, the values of the transferred charge were arranged in the order of $\left|Q^{{\mathrm{NO}}_{2}}\right|>\left|Q^{\mathrm{NO}}\right|>\left|Q^{{\mathrm{SO}}_{2}}\right|>\left|Q^{{\mathrm{NH}}_{3}}\right|>\left|Q^{\mathrm{CO}}\right|>\left|Q^{H_{2}S}\right|>\left|Q^{{\mathrm{CO}}_{2}}\right|>\left|Q^{H_{2}O}\right|>\left|Q^{N_{2}}\right|>\left|Q^{H_{2}}\right|>\left|Q^{{\mathrm{CH}}_{4}}\right|$. CO_2_, CH_4_, H_2_, H_2_O, N_2_, and the host material showed very little charge transfer (< 0.1 e), indicating weak interactions that can be neglected. The remaining six gas molecules can be categorized into two classes, depending on the direction of charge transfer between them and the penta−NiPN monolayer. The first class comprised electron donors, such as H_2_S and NH_3_, which donated electrons (−0.100 *e* and −0.103 *e,* respectively) to the penta−NiPN monolayer, with the corresponding *Q* < 0. In contrast, the second class comprised electron acceptors, such as CO, NO, NO_2_, and SO_2_. For these four gas molecules, the electrons transferred from the host material to the gas molecules during the absorption process. CO and SO_2_ both obtained 0.100 *e* and 0.187 *e*, respectively, which were lower than NO (0.216 *e*) and NO_2_ (0.553 *e*). Therefore, in comparison with other gas molecules, NO and NO_2_ exhibited higher adsorption energy and larger charge transfer during adsorption, suggesting that these gases are more easily adsorbed on the penta−NiPN monolayer, with stronger coupling between them and the host material. Taken together, these results indicate that penta−NiPN monolayer may be a promising material for sensing NO and NO_2_ gases.

**Figure 8:**
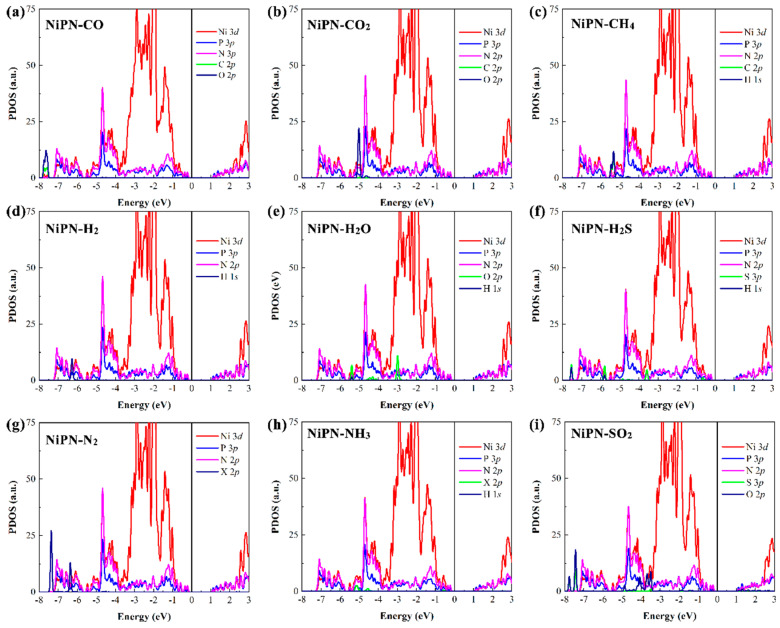
The PDOS (based on HSE06 functional) from the gas molecules are plotted together for the penta−NiPN monolayer with the adsorbed molecules (**a**) CO, (**b**) CO_2_, (**c**) CH_4_, (**d**) H_2_, (**e**) H_2_O, (**f**) H_2_S, (**g**) N_2_, (**h**) NH_3_, and (**i**) SO_2_. Only the spin−up DOS is shown in spin–degenerate systems. The X represents nitrogen atoms.

Last but not least, we further investigated the electronic properties of the penta−NiPN monolayer with adsorption of various gas molecules. Spin polarization was considered in the calculation. We found that, except for NO and NO_2_ adsorbed penta−NiPN monolayers with residual magnetic moments (*M*), all the other adsorbed systems were nonmagnetic. Therefore, we present only the results of NO and NO_2_, considering spin polarization in the latter electronic structures. When nonmagnetic molecules, such as CO, CO_2_, CH_4_, H_2_, H_2_O, H_2_S, N_2_, NH_3_, and SO_2_, were adsorbed, the system remained a nonmagnetic semiconductor with various bandgaps (see Figure 8). The results of PDOS indicated that the orbital hybridization between the gas molecules and the host material was weak or almost non−existent after the adsorption of nine nonmagnetic molecules. Most significantly, the orbital energy levels of gas molecules were primarily in the deep valence band and were distant from the Fermi level (see Figure 8).

**Figure 9:**
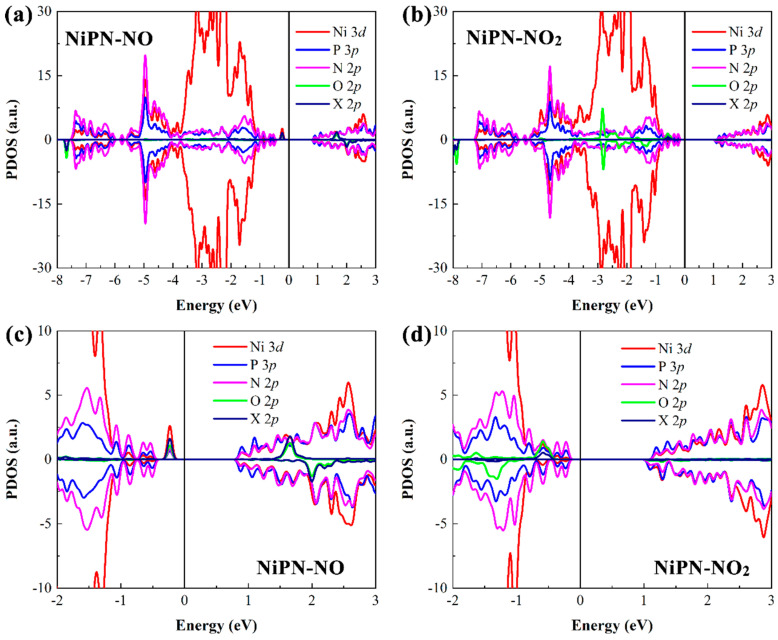
The PDOS (based on HSE06 functional) from the gas molecules are plotted for the penta−NiPN monolayer with the adsorbed molecules (**a**) NO and (**b**) NO_2_. The corresponding enlarged PDOS results of (**c**) NO and (**d**) NO_2_ are given as well. The spin−up and spin−down DOS are shown as positive and negative, respectively. The X represents nitrogen atoms.

Notably, the adsorption of magnetic NO and NO_2_ molecules (1.00 µB) could transform the penta−NiPN monolayer into a magnetic semiconductor. After the adsorption of NO and NO_2_ on the penta−NiPN monolayer, the magnetic moment of NO and NO_2_ was reduced to 0.604 µB and 0.242 µB, respectively. At the same time, the magnetic moment of 0.091 µB and 0.636 µB was introduced into the host material penta−NiPN monolayer, resulting in a total magnetic moment of 0.695 µB and 0.878 µB, respectively, in each system (see Table 3). Therefore, the penta−NiPN monolayer can be electrically and magnetically sensitive to both NO and NO_2_ molecules. In Figure 9, we have plotted the spin−dependent PDOS for the NO and NO_2_ adsorbed systems with various energy ranges, respectively. For the NO adsorbed system, there were electronic states (spin−up DOS) just below the Fermi energy, which contributed to the orbital hybridization between the penta−NiPN monolayer and the NO molecule. Similarly, electron states were introduced into the NO_2_ adsorbed system, but unlike the NO condition, the electron state in NO_2_ was mainly below the conduction band. Clearly, the adsorption of NO and NO_2_ on the surface of the penta−NiPN monolayer induced strong coupling between the gas molecules and the host material. Due to the strong orbital coupling between them, a significant charge transfer occurred. Moreover, the strong adsorption interaction also provided a large magnitude of adsorption energy.

**Figure 10:**
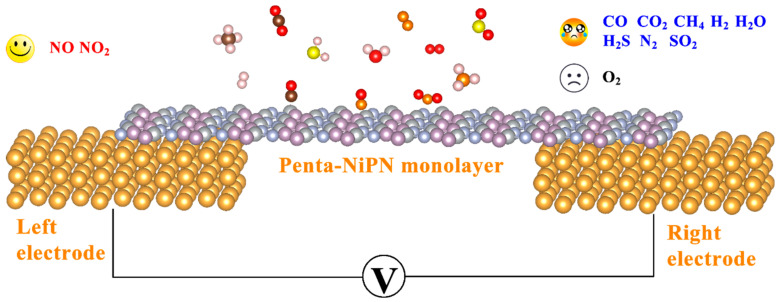
Schematic diagram of gas sensor based on the penta−NiPN monolayer.

In general, the main gas−sensitive mechanisms of 2D materials are surface charge transfer and Schottky barrier (SB) modulation. Upon adsorption of gas molecules on the surface of the host material, the resistance of the material can be shifted. The interactions between gas molecules and 2D materials can either increase or decrease various resistive behaviors, depending on the major charge carriers of the semiconductor and the electron−donating/electron−withdrawing properties of the gas molecules. This resistance change can be further enhanced by creating SBs at the interface between the metal and the semiconductor. In our work, the absorption of NO and NO_2_ by the penta−NiPN monolayer introduced electronic states or impurity levels near the Fermi level or the bottom of the conduction band, directly affecting the electronic transmission properties of the system. This change in electron transport characteristics is manifested as resistance drift in the system. Therefore, gas sensing can be achieved by measuring resistance changes with and without gas adsorption. Figure 10 shows a schematic diagram of a gas sensor based on the penta−NiPN monolayer. When a bias voltage is introduced at the right/left electrode, specific recognition is achieved through the current−voltage curve, reflecting the difference in electronic transmission properties after the adsorption of different gas molecules [21]. However, as the penta−NiPN monolayer serves as the host material for gas sensing, it must be used in an oxygen−free environment. Otherwise, there may be irreversible reactions between the penta−NiPN monolayer and atmospheric oxygen.

## 4. Conclusions

In conclusion, we present a novel ternary penta−NiPN monolayer via first−principles calculations. Our results show that the penta−NiPN monolayer possesses high robust stability, including kinetic, thermodynamic, and mechanical stabilities. We confirmed its quasi−direct bandgap feature with a bandgap value of 1.237 eV based on the hybrid functional HSE06. Its moderate Young’s modulus and Poisson’s ratio indicate that the penta−NiPN monolayer is a promising candidate for flexible electronics. Furthermore, we predicted a hole mobility as high as 1.14 × 10^5^ cm^2^V^−1^s^−1^ in the penta−NiPN monolayer based on deformation potential theory. Moreover, we systematically studied the adsorption properties of 12 common gas molecules (CO, CO_2_, CH_4_, H_2_, H_2_O, H_2_S, N_2_, NO, NO_2_, NH_3_, O_2_, and SO_2_) on the surface of the penta−NiPN monolayer. Our results show that the penta−NiPN monolayer exhibits good selectivity for NO and NO_2_, and has the potential to be used as a sensor for these two gases. Overall, our findings suggest that the penta−NiPN monolayer is a desirable candidate for high−performance electronic devices, as well as NO and NO_2_ gas sensors.

## Figures and Tables

**Table 1:** Calculated lattice constant *a*/*b*, bond length *l*, cohesive energy *E*_coh_, and bandgap *E*_g_ (based on HSE06 functional) of the penta−NiPN monolayer.

Materials	*a* (Å)	*b* (Å)	*l*_Ni−N_ (Å)	*l*_Ni−P_ (Å)	*l*_N−N_/*l*_N−P_/*l*_P−P_ (Å)	*E*_coh_ (eV)	*E*_g_ (eV)
NiPN NiN_2_ [17] NiP_2_ [22]	4.995 4.53 5.55	5.011 4.53 5.55	1.929, 1.910 1.88 −−	2.125, 2.107 −− 2.16	1.605 1.24 2.11	4.55 4.98 4.09	1.237 1.10 0.81

**Table 2:** Calculated m^*^ (unit: *m*_e_), |*E*_il_| (unit: eV), *C*_2D_ (unit: N m^−1^), and *μ*_2D_ (unit: 10^4^ cm^2^V^−1^ s^−1^) for the penta−NiPN monolayer along the *a* and *b* directions.

Materials	Carrier Type	*m_a_* ^*^	*m_b_* ^*^	|*E*_la_|	|*E*_lb_|	*C_a_* ^2D^	*C_b_* ^2D^	*μ*_a_^2D^	*μ_b_* ^2D^
NiPN	Electron	0.38	0.36	2.10	0.85	147.68	146.24	0.51	3.24
	Hole	0.22	0.24	0.74	0.99	147.68	146.24	11.36	5.76
NiP_2_ [22]	Electron	0.106	0.140	5.23	5.23	118.19	118.19	0.71	0.54
	Hole	0.119	0.170	1.53	1.53	118.19	118.19	6.35	4.45

**Table 3:** Calculated adsorption energy (*E*_a_), adsorption distance (*d*), magnetic moment (*M*), and charge transfer (*Q*) between the gas molecules and the penta−NiPN monolayer. Here, “+” and “−” represent gained and lost electrons, respectively.

Gas Molecules	*E*_a_ (eV)	*d* (Å)	$M(\mu_{B}$	*Q* (*e*)
CO CO_2_ CH_4_ H_2_ H_2_O H_2_S N_2_ NO NO_2_ NH_3_ SO_2_	−0.640 −0.184 −0.162 −0.072 −0.272 −0.316 −0.100 −0.751 −1.011 −0.545 −0.445	1.834 3.054 2.661 2.613 2.248 2.210 3.117 1.862 2.065 2.119 2.573	0 0 0 0 0 0 0 0.695 0.878 0 0	+0.100 +0.026 +0.010 +0.011 +0.025 −0.100 +0.015 +0.216 +0.553 −0.103 +0.187

## Data Availability

All data needed to evaluate the conclusions in the paper are present in the paper.
